# Shifting Herbal Knowledge: The Ecological and Cultural Dynamics Behind Plant Use Changes in the Southern Occitan Alps

**DOI:** 10.3390/plants14030367

**Published:** 2025-01-25

**Authors:** Mousaab Alrhmoun, Naji Sulaiman, Andrea Pieroni

**Affiliations:** 1 University of Gastronomic Sciences, Piazza Vittorio Emanuele II 9, 12042 Pollenzo, Italy; a.pieroni@unisg.it; 2 Faculty of Agricultural, Environmental and Food Sciences, Free University of Bolzano, Piazza Università 5, 39100 Bolzano, Italy; 3 Department of Medical Analysis, Tishk International University, Erbil 4001, Iraq

**Keywords:** cultural landscape, ecological drivers, ethnobotany, mountain communities, ethnoecology, temporal changes, traditional ecological knowledge

## Abstract

This study examines changes in medicinal and wild food plant knowledge in the Alpine Southern Occitan area, focusing on temporal and cultural shifts. Drawing from ethnobotanical data from the Maira Valley (1970, 2022), Stura Valley (2004), and Grana Valley (2011), we explore dynamics in plant use and how they may have been generated by ecological, socio-economic, and cultural changes. A total of 308 plant taxa were recorded, with notable declines in Asteraceae and Lamiaceae utilizations by 2022, suggesting a remarkable erosion of traditional ecological knowledge (TEK). Conversely, the use of families like Brassicaceae and Amaranthaceae increased, possibly due to socio-economic factors. This study also found in recent years a decrease in medicinal plant uses and a greater reliance on food system-related plants. Logistic regression models highlighted altitude (600–1600 masl) as a key factor in plant diversity use, with older participants showing more diverse ethnobotanical knowledge. This study underscores the resilience and transformation of plant knowledge in response to socio-economic and environmental changes in the Alpine area.

## 1. Introduction

Traditional ecological knowledge (TEK) is vital for local communities and their well-being. TEK has been a focal point of research within the ethnobiological field, and recent developments have witnessed a shift towards a diachronic approach that emphasizes the historical evolution of these corpora of knowledge [1]. The Alpine Southern Occitan area, nestled between the Western Alps and the borderlands of Italy and France, represents a unique convergence of cultural and ecological characteristics [2]. Known for its breathtaking alpine landscapes, this region is defined by a rich history of small-scale agricultural practices, traditional ecological knowledge (TEK), the survival of the local Occitan language, and close-knit communities that have adapted to the region’s challenging environment [3,4,5]. At altitudes ranging from 600 to 3000 m, the area is characterized by a distinct climate marked by moderate to cold temperatures and a significant seasonal variation in precipitation [2,6]. This ecological diversity has shaped a wealth of botanical knowledge passed down through generations, contributing to the local identity of the region’s inhabitants. Over the years, however, this knowledge has been increasingly threatened by various factors, including socio-economic shifts, migration, changing environmental conditions, and land use alterations that especially started in the Sixties and Seventies of the past Century with the move of many locals to urban centers for labour. These influences collectively contribute to the erosion or transformation of TEK, making it essential to examine the broader context of these changes in understanding the region’s ecological knowledge dynamics [7,8].

Traditional ecological knowledge (TEK) refers to the body of knowledge, practices, and beliefs about the relationship of living beings (humans, animals, and plants) with their environment [9]. This knowledge system was once integral to daily life in the Alpine Southern Occitan area, influencing everything from agricultural practices to health, spirituality, and social organization [10]. However, the erosion of TEK has become a critical issue as the region faces a phenomenon known as the “Hysteresis Effect”, the idea that, once knowledge systems are lost, they may be difficult or impossible to recover, even when conditions for its re-establishment return [11]. This effect is compounded by the social and economic transformations that the region has experienced in recent decades, which have led to the displacement or disappearance of key cultural practices and the disintegration of long-standing knowledge systems [12,13].

Several factors influence the persistence and decline of TEK in this region, and these factors are often interconnected [2]. Climate change, particularly shifts in temperature and precipitation patterns, plays a pivotal role in altering plant ecosystems, which in turn influences the knowledge that local communities maintain about these plants [9,14]. Increases in average temperatures, changing precipitation patterns, and the unpredictability of seasonal weather can directly affect the availability of plant species that were once integral to the region’s agricultural and medicinal practices [7,15]. This, in combination with altitude, may further exacerbate the decline or transformation of plant-based knowledge. Higher altitudes experience more pronounced changes in climate, with species adapted to specific temperature and moisture conditions potentially becoming rarer or migrating, altering the local flora and the knowledge tied to it [6,16,17].

In addition to environmental factors, land use changes and the reduction in agricultural activity have contributed significantly to the erosion of traditional knowledge [18]. Over the decades, farming practices have been reduced or altered due to economic pressures, population decline due to urbanization, leading to a shift in how people interact with the land. This shift also has implications for the cultivated or harvested plants, which are no longer as deeply integrated into daily life. As land abandonment increases, particularly in high-altitude areas once used for traditional agro-pastoralism, the continuity of TEK is disrupted [19]. A recent study documented that temporal shifts in plant usage have occurred in neighboring Alpine Ubaye and Bellino Valleys [20]. The study suggests that this shift reflects broader cultural, ecological, and socio-economic changes, underscoring the importance of preserving biodiversity and traditional knowledge amidst ongoing environmental and societal shifts.

Cultural factors such as modernization, changes in economic levels, and the effects of migration also shape the way knowledge is passed down. As younger generations leave for urban centers in search of better education and job opportunities, the transmission of traditional knowledge becomes less consistent [21]. The influx of new residents, including migrants, seasonal workers, and tourists, further complicates the local knowledge landscape, introducing new plant knowledge, practices, and cultural exchanges but also contributing to the dilution of TEK held by long-established inhabitants.

Notably, few studies have addressed the risk factors influencing the decline or transformation of ethnobotanical knowledge over extended periods. While some global studies are tracking these changes, very few have examined the long-term effects (over 50 years) on ethnobotanical taxa in such detail [1,3,7,12].

These studies are critical, as they provide insight into the possible resilience of traditional plant knowledge systems and the broader ecological and cultural shifts that affect them. These rare longitudinal studies highlight how risk factors such as climate change, land use modifications, migration, and socio-economic transitions can significantly alter the presence, utilization, and transmission of local plant knowledge across generations [1,12].

This study aims to explore the interrelation between the loss of traditional ecological knowledge (TEK) and the emergence of new forms of ecological and cultural knowledge over time in the Alpine Southern Occitan area. By examining historical and contemporary data on local plant knowledge, this research seeks to better understand the mechanisms through which knowledge is lost, adapted, or revitalized. Through a multi-generational lens, this study will assess how changing economic, social, and environmental conditions influence knowledge systems and how communities negotiate their relationship with their natural environment.

## 2. Results

### 2.1. Plant Diversity Across the Southern Occitan Alps

In the studies analyzed, a total of 308 botanical taxa were identified in the study area (Maira, Grana, and Stura valleys), 112 in the Rovera (1982) study [22], 90 in Musset and Dore (2004) [23], 86 and 20 in our collected data in 2011 and 2022, respectively. These recent collected data reveal different trends in plant family usage, with 50 families identified in Rovera et al. 1982 [22] 38 in Musset and Dore, 2004 [23], and 43 and 15 in our collected data in 2011 and 2022, respectively.

Table 1 presents the usage of plant parts across four studies: Rovera et al. [22], Musset and Dore [23], and the unpublished data of our research group collected in 2011 and 2022 highlighting the temporal shifts in botanical taxa and family utilization. The most prominent families identified in Rovera et al. [22] include Asteraceae, Lamiaceae, Rosaceae, Apiaceae, Violaceae, Amaryllidaceae, Fabaceae, Malvaceae, Oleaceae, Ranunculaceae*,* and Urticaceae. Musset and Dore [23] follow a similar pattern with the frequent appearance of Asteraceae, Lamiaceae, Rosaceae, Apiaceae, Pinaceae, Fabaceae, and Polygonaceae. Our collected data in 2011 highlight families such as Asteraceae, Lamiaceae, Brassicaceae, Liliaceae, Apiaceae, Campanulaceae, and Rosaceae. On the other hand, our collected data in 2022 narrow significantly, with only Asteraceae and Lamiaceae remaining as the most prominent families.

**Figure 1:**
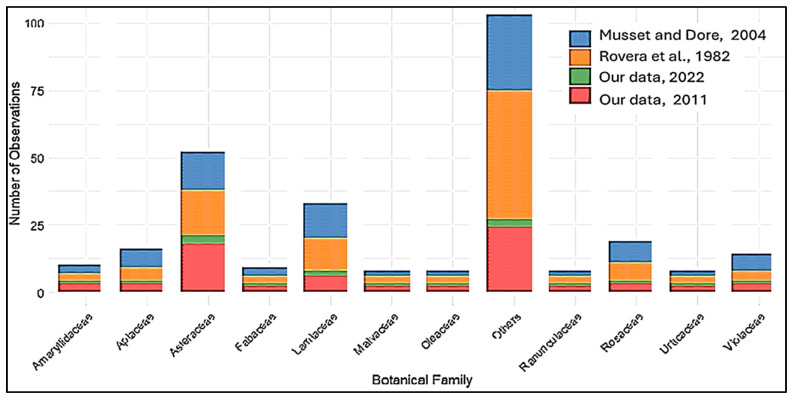
Number of reported botanical plants in the past and present studies in the data area [22,23].

The analysis of plant families reveals notable shifts over time, reflecting changing ecological and cultural roles. Families such as Asteraceae and Lamiaceae have maintained a continuous presence across all studies, though with a marked decline in the more recent data. For example, Asteraceae was cited in 1982 and 2004, but only in 2022 was its presence notably reduced. Similarly, Lamiaceae decreased significantly from 1982 to 2022 (Figure 1).

Certain families, such as Amaranthaceae, emerge with increasing frequency in later studies, indicating a shift in plant use, possibly influenced by socio-economic factors like migration and urbanization. However, these families were not consistently present across all datasets, and their inclusion in Figure 1 is limited to the years where they were observed.

The “Others” category, which includes less frequently cited families, has also sharply declined from 1982 to 2022, reflecting a narrowing of plant diversity within local knowledge systems (Figure 1).

**Figure 2:**
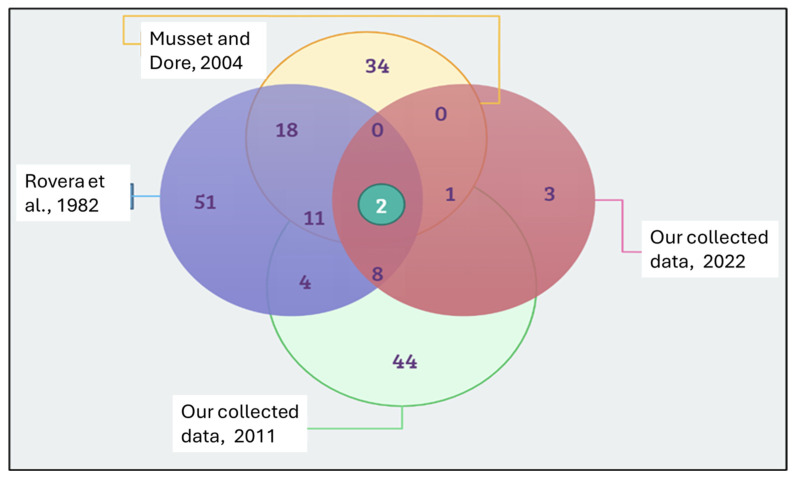
Genera overlapping within the present data and previous studies conducted in the region from 1970 to 2022 [22,23].

The results presented in Figure 2 demonstrate significant variation in the documented plant genera across different temporal contexts, reflecting changes in both the number and frequency of ethnobotanical knowledge over time. Earlier studies, such as Rovera et al. [22] and Musset and Dore [23], record a wide range of genera, with Rovera et al. [22] alone documenting 51 genera and Musset and Dore contributing 34. These studies highlight frequently cited plants such as *Achillea millefolium*, *Artemisia absinthium.*, and *Calendula officinalis,* which represent long-standing staples of traditional practices.

In contrast, more recently collected data, such as (2022) and (2011), report fewer genera, with 2022 data listing only 3 genera and data (2011) documenting 44. Notable among the recent additions are *Allium ursinum* and *Silybum marianum*, which appear with increasing frequency, suggesting their growing prominence due to changing ecological or cultural factors. However, despite this overall reduction in diversity, some botanical plants, such as *Pimpinella anisum* and *Sambucus nigra*, persist across all four studies, reflecting their sustained importance in ethnobotanical traditions.

Frequency analysis reveals that some botanical plants, like *Pimpinella anisum.*, appear in up to 11 combinations of studies, demonstrating their widespread and enduring utility. Others, like *Melissa officinalis*, are mentioned in only one combination, highlighting their more specialized or localized relevance. This fluctuation in frequency underscores the dynamic nature of ethnobotanical knowledge, where cultural preferences, environmental changes, and practical needs shape the prominence of certain genera over time.

### 2.2. Shifts in Traditional Plant Knowledge, Usage, and Biodiversity Across Study Sites in the Southern Occitan Alps

**Figure 3:**
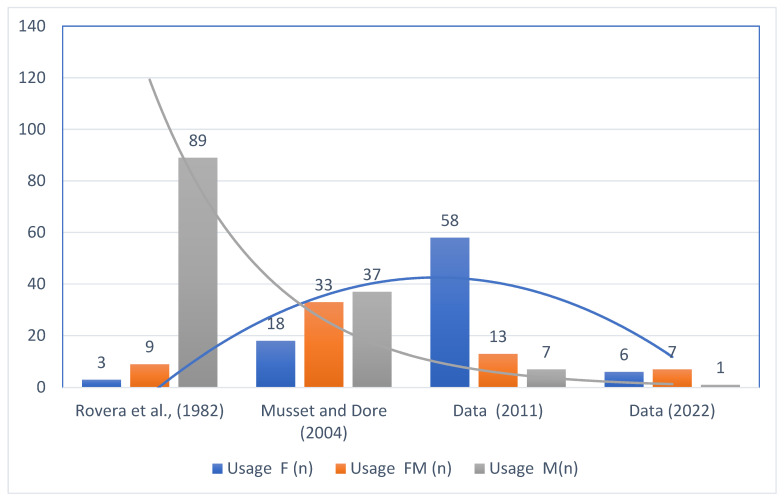
The distribution of plant part usage ((F) food, (FM) both food and medicinal, and (M) medicinal) across the studies ([22,23]), and our collected data in 2011, and 2022.

The results of this study revealed notable shifts in plant utilization over time, which can be compared to the patterns observed in Rovera’s study [22], the majority of plant parts used were medicinal (88.1%), food and medicinal (8.9%), and food (3.0%) utilized less frequently (Figure 3). This indicates a primary focus on medicinal applications during this period. By contrast, Musset and Dore (2004) showed a more balanced distribution, with 42.0% of plant parts used for medicinal purposes, 37.5% for food and medicinal, and 20.5% for food. This shift suggests a growing emphasis on fruits and flowers alongside medicinal uses. Our collected data (2011) indicate a shift towards a food-centric approach, with 74.4% of plant parts used for food purposes, while smaller proportions were used for both food and medicinal purposes or solely for medicinal purposes (16.7% and 9.0%, respectively) (Figure 3). Finally, our data (2022) highlight a significant increase in the use of food, which accounted for 50.0% of the plant parts, followed by food and medicinal use (42.9%) and medicinal use (7.1%). This recent trend points to a shift towards greater food utilization, likely reflecting changes in cultural practices or available plant resources. These temporal shifts in plant part usage underscore the evolving roles of plant species, influenced by ecological, cultural, and socio-economic factors over the decades.

Overall, these findings indicate a notable shift in plant part usage over time. While past studies, particularly from Rovera (1982), focused heavily on medicinal species, more recent data, especially the data (2011) and collected data (2022), show an increasing trend in the use of plants for fodder and food purposes. This shift may reflect changing agricultural practices, environmental conditions, and evolving cultural preferences in the use of plant species. The overall trend suggests that, while medicinal uses remain important, there has been a marked increase in the functional diversity of plants, particularly in terms of their role in animal husbandry and food resources. This shift indicates broader changes in socio-economic and ecological contexts over the years.

The reason for the medicinal plant decrease may be twofold: (a) medicinal plants were especially needed and used when public health care was less widespread and accessible (see the high number of medicinal taxa in a study published in 1982); and (b) medicinal species were collected in ecological areas insisting on the spread and robust forestry and shepherding activities; these activities have basically disappeared nowadays.

The opposite trend of food plant foraging could be instead explicable with the huge interest that, in the past two decades, foraging has (re-)gained in the data area and in all throughout Northern Italy, possibly bringing also new food plant-centered practices.

**Figure 4:**
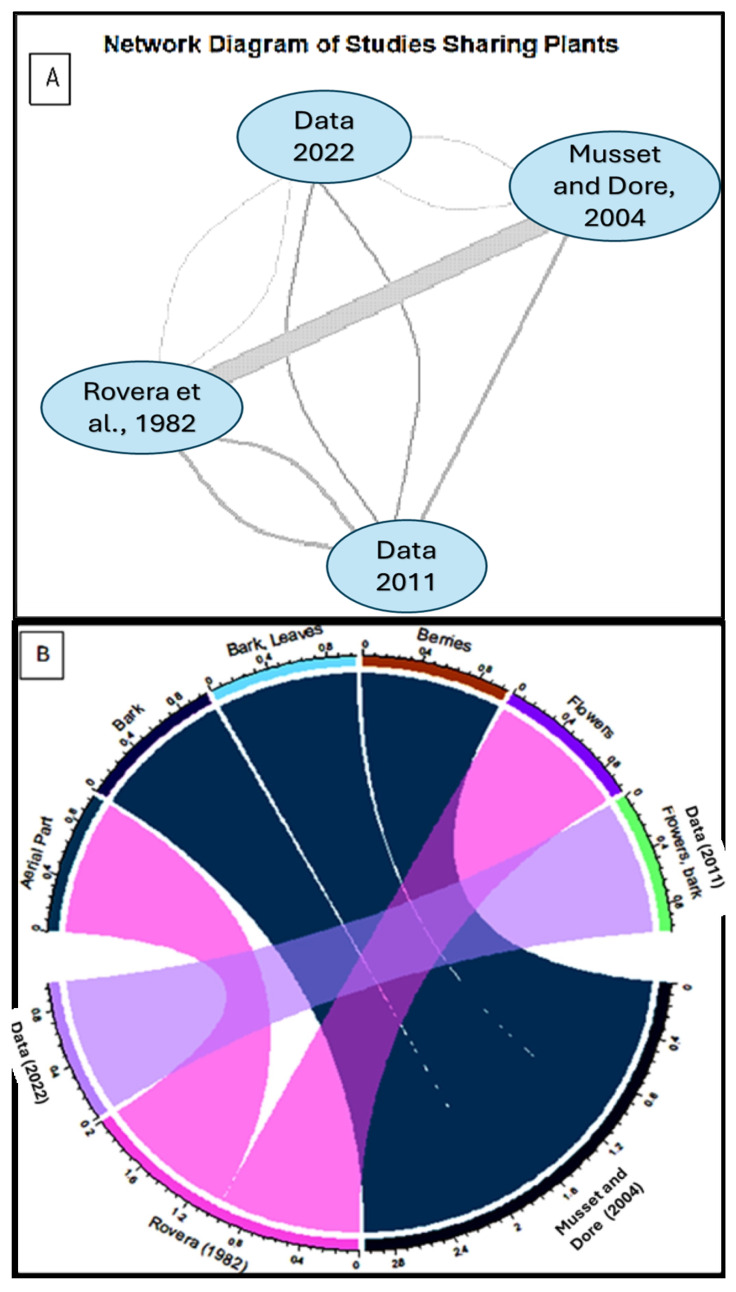
(**A**) The interconnectedness of plant studies and the overlap in plant parts examined across different studies [22,23]. (**B**) The chord diagram confirms the dynamic interplay between all the studies over time.

The network diagram (Figure 4A) reveals that the collected data in 2022 shares plant parts with Musset and Dore 2004, Rovera 1982, and data in 2011. Similarly, Musset and Dore 2004 and Rovera 1982 also share plant parts with our collected data in 2011. This interconnectedness suggests potential connection based on the same region area and mountain community among these studies, especially the same ecosystems and per consequence that will confirm their variation based on risk factors. The chord diagram (Figure 4B) confirms the dynamic interplay between the studies over time, despite their focus on the same region and valleys.

Earlier studies, such as Rovera et al. (1982), may have prioritized more traditional plant uses, focusing on parts like aerial parts and flowers. In contrast, more recent studies, like our collected data in 2022 and 2011, seem to explore a broader range of plant parts, potentially reflecting evolving research interests and methodologies (Figure 4B).

Understanding these shifts and overlaps is crucial for a comprehensive understanding of plant use and ecology in the region.

**Figure 5:**
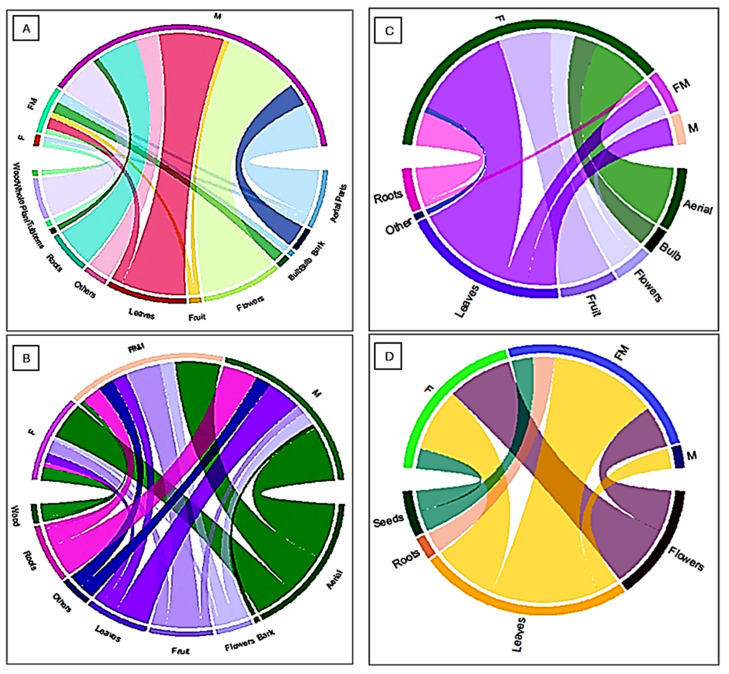
The chord diagram between all the usage and used parts in each data over time: (**A**) Rovera et al. [22], (**B**) Musset and Dore [23], (**C**) our collected data (2011), and (**D**) data (2022).

The chord diagrams (Figure 5) provide a visual representation of the overlap in plant parts used for medicinal and food purposes across different studies. The thickness of the ribbons connecting the plant parts indicates the extent of overlap. For instance, the significant overlap between “Bark” and “Leaves” in both diagrams suggests that these plant parts are commonly used for both medicinal and food purposes. This finding aligns with traditional knowledge systems where various plant parts are utilized for diverse applications. Similarly, the overlap between “Flowers” and “Fruit” indicates that these reproductive plant parts are often used in both culinary and medicinal practices. This overlap could be attributed to their potential medicinal properties, such as anti-inflammatory or antioxidant effects.

On the other hand, plant parts like “Wood” and “Roots” show less overlap with other categories, suggesting a more specialized use in either medicine or food. This could be due to the structural properties and specific chemical compounds, like tannins, alkaloids, and flavonoids, found in wood and roots. These compounds, with antimicrobial, anti-inflammatory, or antioxidant properties, make these plant parts more suitable for targeted therapeutic uses. Additionally, their dense nature and secondary metabolites limit their versatility, confining them to specific roles in traditional medicine or food.

When analyzing the data separately and connecting it with the tier conditions, the different aspects of the interconnection between the plant parts and their uses become more apparent.

Diagram A (Figure 5), representing Rovera et al. [22], reveals a more pronounced overlap between “Bark” and “Leaves,” indicating a strong association of these plant parts with both medicinal and food applications. Additionally, the overlap between “Flowers” and “Fruit” is significant, suggesting their versatile use in various cultural practices.

Diagram B, which corresponds to Musset and Dore [23] shows a more balanced distribution of overlap across different plant parts. While “Flowers” and “Bark” still exhibit a notable overlap, other plant parts like “Leaves” and “Fruit” show a more moderate level of overlap. This implies a potentially wider range of plant parts used for both medicinal and food purposes in the context represented by this study. Chord diagram C, based on our collected data (2011), further highlights the overlap between medicinal and food uses of different plant parts. It illustrates a diverse range of plant parts utilized for various purposes, showing how cultural practices have expanded the number of plant parts used for different applications.

Finally, chord diagram D, representing our collected data (2022), offers insights into the continued use and overlap of plant parts, with some newer trends emerging in the overlap patterns. Like diagram C, it reflects a broad utilization of plant parts but with some shift in preferences based on more recent ecological or cultural influences. Each diagram illustrates the evolving relationship between plant parts and their medicinal and food uses, emphasizing the dynamic and interconnected nature of ethnobotanical knowledge across different time periods (Figure 5).

### 2.3. Patterns, Similarities, and Knowledge Dynamics: A Comparative Analysis Through Heatmaps, Dendrograms, and Principal Component Analysis (PCA)

**Figure 6:**
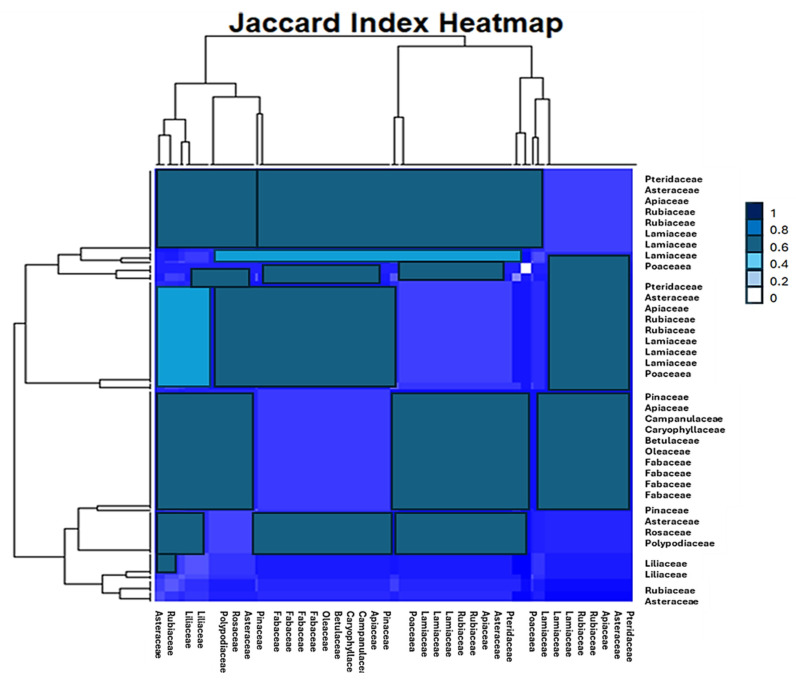
The heatmap illustrates the Jaccard Index, measuring the similarity between plant species compositions across various families. Values range from 0 (no overlap) to 1 (complete similarity), represented by a gradient from dark blue (low similarity) to cyan (high similarity). The hierarchical clustering on both axes highlights groups of plant families with shared characteristics, as shown by closely aligned branches in the dendrogram.

Notable clusters include families such as Apiaceae and Asteraceae, which exhibit significant overlap, while families like Liliaceae and Rubiaceae appear more distinct with lower similarity, as indicated by darker hues (Figure 6). Figure 6 emphasizes compositional relationships and biodiversity patterns, providing insights into ecological associations and the distinctiveness of certain plant families. The diagonal symmetry confirms the consistency of the Jaccard Index in reflecting these relationships.

The dendrograms on the sides of the heatmap represent the hierarchical clustering of the data sets. Similar data sets are grouped, forming clusters. The height of the branches in the dendrogram reflects the similarity between the clusters.

Overall, the heatmap provides a visual representation of the relationships between the data sets, allowing you to identify groups of similar data sets and understand the patterns of similarity and dissimilarity.

**Figure 7:**
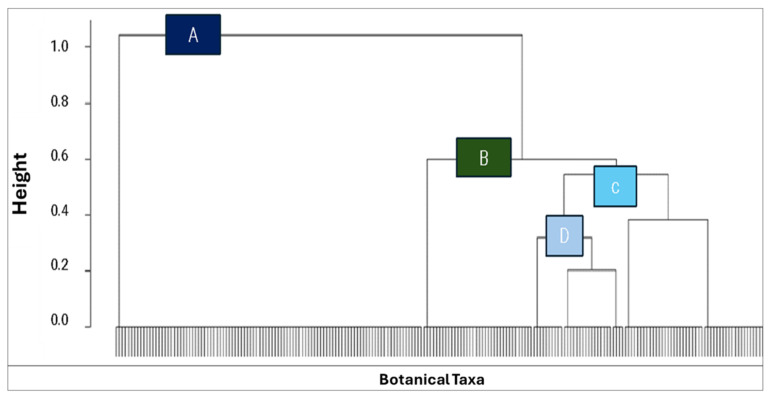
A dendrogram is a tree-like diagram used to visualize hierarchical relationships between data studies labeled (**A**) Rovera et al. [22], (**B**) Musset and Dore [23], (**C**) our data (2011), and (**D**) our data (2022).

**Figure 8:**
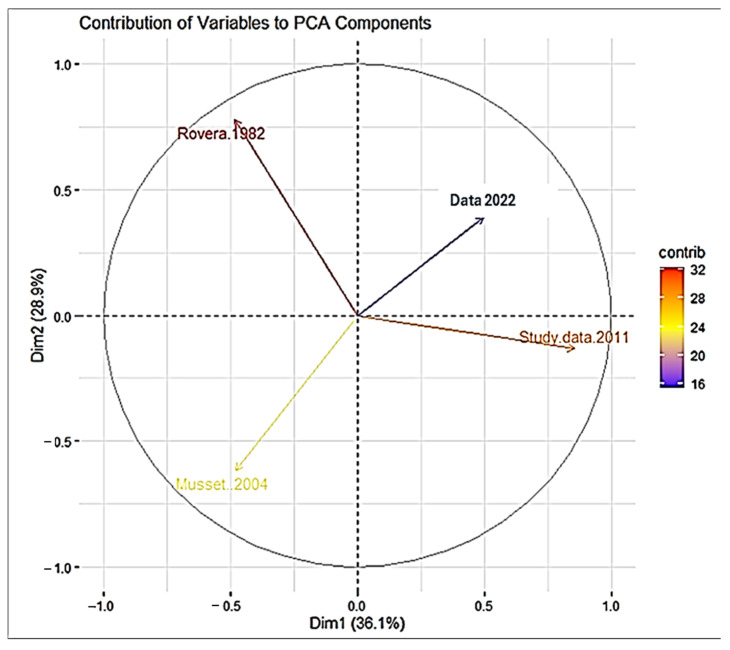
The biplot visualizes the relationships between the four studies based on their contribution to the principal components. The studies can be clustered into two groups: Cluster 1 comprising Rovera et al. [22] and Musset and Dore [23], and Cluster 2 including Data 2011 and Data 2022.

The dendrogram (Figure 7) illustrates the hierarchical clustering of four studies, revealing distinct patterns of similarity and dissimilarity. Data A (Rovera et al. [22]) emerges as the most unique, forming an independent cluster. Data B (Musset and Dore [23]) shares some similarities with A but also exhibits distinct characteristics, placing it in a separate cluster. In contrast, Studies C (Data 2011) and D (Data 2022) demonstrate a high degree of similarity, forming a closely related cluster. This suggests that the latter two studies’ data may share common botanical taxa focus or families and ecosystem factors, differentiating them from the earlier studies, and this was confirmed by the PCA (Figure 8).

The first principal component (PC1) separates these clusters, indicating that it captures the primary source of variation between the studies. Rovera et al. [22] and Musset and Dore [23] exhibit similar patterns of variation, In contrast, our collected Data 2011 and 2022 demonstrate distinct patterns (Figure 8).

The length of the arrows in the biplot illustrates the impact of each study on the principal components to the principal components. Rovera et al. [22] have a greater influence on the overall variation in the data. By analyzing the biplot, we can gain insights into the similarities and differences between the studies and identify potential trends or patterns in their research.

### 2.4. Factors Influencing Botanical Diversity: Insights from Logistic Regression Analysis

To understand and complete our version of the results a logistic model was developed.

The results (Table 2) revealed that altitude was a significant predictor, with the 600–1600 m range showing the strongest effect (odds ratio = 2.22, *p* = 0.002). This supports the findings of Rovera et al. [22] where the highest number of taxa (112) was observed under similar altitude conditions. The temperature categories (5 to 12 °C and 7 to 13 °C) did not show a significant influence on botanical diversity, with a *p*-value above 0.05, suggesting that temperature may have a weaker effect than altitude in shaping plant diversity. Precipitation was also not a significant factor, with *p*-values of 0.511 and 0.151 for the 1400–1600 mm and 1200–1400 mm categories, respectively. The age range of participants showed a significant effect, with individuals in the 71–75 years category reporting a higher number of botanical taxa (odds ratio = 2.34, *p* = 0.004), indicating that older participants may possess more knowledge about the local flora. Finally, data sources showed a marginal effect, with interviews slightly more informative (odds ratio = 1.82, *p* = 0.090) compared to herbarium data, although this result was not statistically significant at the 0.05 level (Table 2). These findings underscore the importance of altitude and age in understanding the diversity of botanical taxa while also highlighting the relative influence of direct interviews over other data sources in capturing plant diversity.

## 3. Discussion

### 3.1. Resilience and Change in Plant Use in the Southern Occitan Alps

The analysis of ethnobotanical data from the Alpine Southern Occitan area reveals a notable reduction in plant species diversity over the past few decades. When comparing the four studies spanning from 1982 to 2022, it is evident that the number of documented taxa has decreased significantly. While the Rovera et al. [22] and Musset and Dore [23] studies cataloged a wide variety of species, including many medicinal plants such as *Achillea millefolium* and *Artemisia absinthium*, the later studies (particularly our collected data in 2022) show a marked decline in the number of species reported.

As noted, there has been a dramatic decrease in the number of herbs actively used in the region. Herbs such as *Achillea*, *Artemisia*, *Veronica,* and *Viola* genera, which were once key components of local diets and medicinal practices, have seen their usage diminish significantly. These genera are now considered uncommon, and, in many cases, are no longer present in the local herbal market. This decline can be linked to the diminishing number of people who still engage with the natural environment daily [24,25]. The role of these herbs, which once had medicinal and culinary applications, is now largely forgotten or relegated to anecdotal references in older generations. The shift away from traditional ecological practices has led to the loss of a deep knowledge base surrounding these plants, which had once been part of the fabric of daily life [10,16].

Another significant trend revealed by the results is the changing emphasis on different plant parts over time. The earlier studies [22] and [23], show a strong emphasis on the medicinal uses of plants, with flowers, leaves, and roots as the most commonly used parts. However, in more recently collected data, such as Data 2011 and (2022), the focus has shifted towards food-related uses, particularly fruits. This shift reflects changing dietary patterns in the region, where food sources have become more central to local consumption [26,27]. The increased use of fruits, such as those from Rubus and Malus species, mirrors broader trends in the local food culture, likely influenced by shifts in agriculture, food security concerns, and a growing preference for locally sourced, seasonal foods [28,29]. This change is especially notable in our collected data in (2022), where fruits comprise 50% of plant part usage. This suggests that, while medicinal plant use declines, plant species that contribute to food security and nutritional needs are becoming more significant in the cultural practices of the region.

### 3.2. Ecological and Socio-Economic Drivers of Plant Knowledge and Diversity

The role of ecological factors in shaping plant knowledge and diversity was also explored. One key finding is the strong relationship between altitude and plant diversity. The studies consistently show that higher altitudes are associated with greater plant diversity, particularly for medicinal and food-related species. This result aligns with Rovera’s findings [22], which reported the highest diversity of plants at altitudes between 600 and 1600 masl. The environmental conditions of these higher altitudes likely foster a wider range of plant species, providing diverse resources for the local population [30]. Interestingly, the analysis revealed no significant correlation between plant diversity and temperature or precipitation categories, suggesting that altitude is a more significant factor in shaping plant diversity than climate alone [31]. This finding emphasizes the unique ecological conditions of the Alpine Southern Occitan region, where altitude appears to be a key determinant of both plant diversity and the extent of traditional plant knowledge.

The socio-economic changes in the region have played a major role in altering plant use patterns. The decrease in medicinal plant knowledge can be seen as part of a broader trend toward modernization and the decline of traditional farming and foraging practices [32]. The younger generations in the region are less likely to engage in traditional agricultural practices and are more reliant on commercial food systems, which has contributed to the shift away from plant usage [18,30]. The increased use of certain plant families, such as Brassicaceae and Amaranthaceae, suggests that external factors, including changes in food security and agricultural practices, have influenced plant selection. These shifts could also reflect the resurgence of foraging in privalently anthropogenic environments, while the pastoralist landscape has been dramatically abandoned and the introduction of new (wild) food plant uses may have displaced older, more traditional species [33]. The reduction in the use of some plant families, such as Asteraceae, further points to the impact of changes in land management and also urbanization [34].

Despite this decline, there remains an opportunity to revitalize the use of these herbs, particularly in innovative food and beverage sectors. The use of herbs such as *Achillea*, *Artemisia*, *Veronica*, and *Viola* could play a significant role in the development of novel food products. These herbs, which are not yet widely available on the herbal market, hold untapped potential for sustainable, innovative food and beverage applications [35,36]. They could be incorporated into health-conscious, eco-friendly products that align with current trends towards natural and local ingredients. Exploring the culinary and medicinal potential of these plants could reinvigorate interest in traditional plant knowledge, offering both ecological and economic benefits by reintroducing these species into contemporary markets [37].

The revival of interest in these plants could also help bridge the knowledge gap created by the loss of TEK. By integrating these herbs into modern products, there is an opportunity to reconnect people with their environmental heritage, fostering a renewed relationship with local plants [24,38]. This process could help counteract the so-called Hysteresis Effect by reintegrating forgotten knowledge into the cultural landscape, even in the absence of daily farming-related engagement with nature that was the pillar of rural communities in the Alps until the 1970s. These plants, once critical to rural livelihoods, could once again play a role in the ecological and cultural revitalization of the region.

The findings from this data provide valuable insights into the changing relationship between local communities and their plant resources. Efforts are urgently needed to preserve traditional ecological knowledge, primarily through community-based initiatives. Future research should focus on revitalising plant knowledge by engaging local communities in documentation and education programs that emphasise the importance of plants for both cultural and ecological sustainability. Longitudinal studies that explore the relationship between socio-economic shifts, environmental changes, and plant knowledge would be valuable in further understanding the factors contributing to the erosion of TEK. Furthermore, research that includes a broader range of ecological zones and socio-economic contexts could provide a more comprehensive view of the forces driving these shifts across the region.

### 3.3. Limitations of This Study

Several factors may have limited this study. First, as in every historical-ethnobotanical comparison, the exact field methods adopted by diverse researchers at different times could have been slightly different, posing an accent more or less pronounced on the keywords “herbs” (vs “non-herbal” taxa) or on “food” vs “medicine”-centred plant utilisations. Additionally, the study focused on three specific portions of valleys within a broad Italian Western Alpine region in the Southern Occitan linguistic area, which may not represent the entire diversity of plant knowledge of other Occitan or Western Alpine regions. The historical context of plant use is also challenging, as the decline of traditional practices over decades may have led to gaps in knowledge: participant memory and subjective interpretations of plant usage could have influenced the accuracy of the data. Moreover, the data do not extensively explore other factors, such as environmental changes or dynamics in agricultural practices, that might affect plant use. Future research should address these limitations by broadening the scope, including younger generations, and exploring additional ecological and socio-economic factors.

## 4. Materials and Methods

### 4.1. Study Area

**Figure 9:**
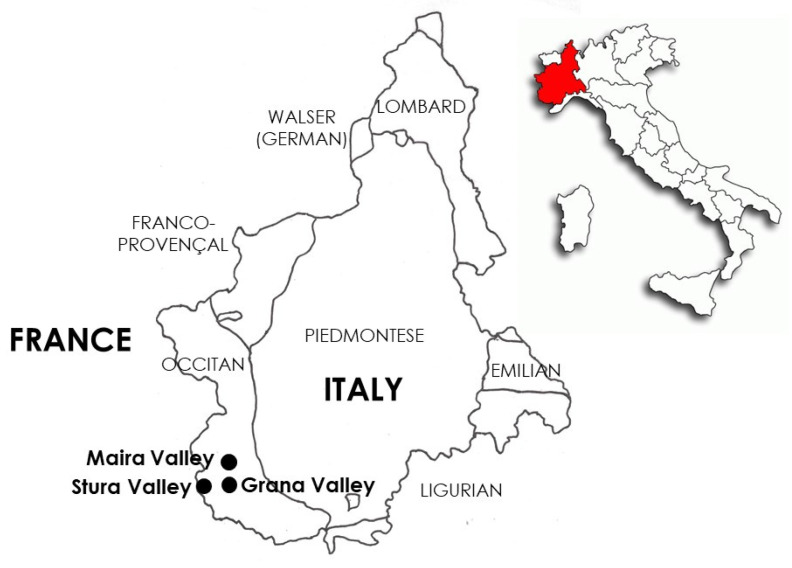
Linguistic map of Piedmont (NW Italy) and the three considered Alpine Southern Occitan valleys (Maira, Stura, and Grana), nestled in the borderlands of Italy and France.

The Alpine Southern Occitan area (Maira, Stura, and Grana valleys), nestled between the Western Alps and the borderlands of Italy and France (Figure 9), is a region steeped in a rich history of both ecological and cultural significance [39]. This area has long been home to communities that have developed unique agricultural and medicinal practices suited to the challenging alpine environment. At altitudes ranging from 600 to 3000 m, the region is defined by a distinct climate, marked by moderate to cold temperatures and significant seasonal variations in precipitation, creating a diverse range of ecological zones [10]. These varying altitudes have resulted in an exceptional variety of plant species, many of which are deeply embedded in the traditional practices of the local communities. The history of the region is characterized by a longstanding reliance on farming, pastoralism, and local plant knowledge, which has shaped both the cultural and ecological landscapes. Over time, these communities have developed intricate systems of ecological knowledge that integrate plant use with the broader cultural and spiritual practices of the region.

The region’s people, traditionally organized in small, close-knit communities, have a strong sense of identity rooted in the land they cultivate and the ecological resources they manage. This connection to the land is reflected in the way traditional knowledge has been passed down through generations, often through familial lines and communal teachings. The transmission of knowledge was primarily oral, with elders passing down plant-based wisdom related to medicinal, culinary, and agricultural practices. This knowledge was not only practical but deeply tied to local cosmologies and worldviews, which held a spiritual and ethical connection to nature [10,40]. For instance, certain plants were considered sacred, and their use was governed by strict cultural protocols that ensured sustainable harvesting and respect for the natural world. However, the interplay between ecological conditions, such as the region’s altitude and climate, and cultural practices has been increasingly disrupted by socio-economic shifts. The pressures of modernization, economic changes, and out-migration have altered the way younger generations relate to traditional practices, with many abandoning them in favor of urban life and more standardized agricultural techniques.

### 4.2. Fieldwork and Data Collection

For this comparative data, the fieldwork spans several decades, from 1982 to 2022, and includes both historical and contemporary data on local plant knowledge. The data collection process involved interviewing individuals from a range of professions, including farmers, restaurateurs, and workers in the tourism industry (Table 3). These interviews captured the changing dynamics of plant use and ecological knowledge, shedding light on how the local plant knowledge system has evolved in response to broader socio-economic and environmental shifts.

**Figure 10:**
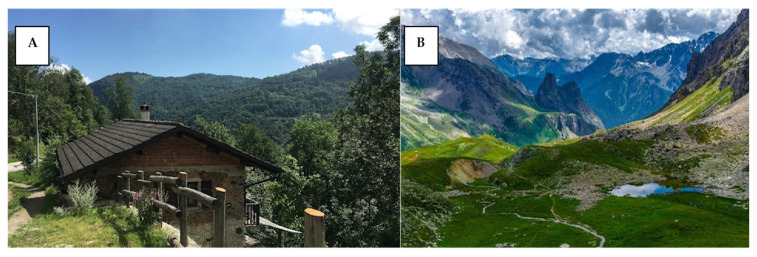
Natural landscape in the Valle Grana (**A**) and Maria (**B**) (Photo: Site Maira Valley, Italy).

In addition to interviews, field observations were conducted to record plant species used, with an emphasis on how climate change and land use alterations that have impacted plant distribution and abundance at different altitudes (Figure 10) will give a view on this altitude and the natural landscape. These observations allow for an in-depth understanding of how ecological changes, such as shifts in temperature or precipitation patterns, influence local plant knowledge and practices. Moreover, the influx of migrants into the region has brought new plant knowledge and altered traditional practices, further complicating the region’s ethnobotanical landscape. Plant specimens were collected, identified, and deposited in a recognized herbarium during previous ethnobotanical fieldwork conducted by some of the authors in the contiguous areas of the Western Alps [10,40]. Verbal consent was always obtained from the data participants, following the Code of Ethics of the International Society of Ethnobiology [41].

### 4.3. Data Analysis

The analysis of the collected data will be conducted using both SAS 9.4 and R v4.4.2 to explore the relationships between ecological and cultural factors influencing plant knowledge and its transmission. Statistical techniques such as PCA (Principal Component Analysis) will be used to reduce the complexity of the data and identify key ecological and cultural variables that explain variations in plant knowledge across the region. Cluster analysis will also be employed to group plant species based on their cultural and ecological significance, revealing patterns in how these species are used and shared among different communities. In addition, Redundancy Analysis (RDA) will be utilized to examine the relationship between ecological factors such as temperature, precipitation, altitude, and plant species distribution. This will allow the data to assess how environmental conditions influence the preservation or loss of traditional plant knowledge.

Furthermore, a logistic regression model [42] was applied to assess the relationship between the botanical taxa presence and various ecological and socio-economic factors. The explanatory variables included altitude, temperature, precipitation, age range, and data source. The botanical taxa data were treated as a binary outcome, indicating the presence or absence of species in different environmental conditions. The model results suggest that age range and altitude are significant predictors of botanical taxa presence.

The general form of the logistic regression model is as follows:

$$Logit\left(p\right)=lnln\;\left(\frac{p}{1-P}\right)=\beta 0+\overset{n}{\underset{i=1}{\sum }}1\beta iXi$$

where

- *p* is the probability of the event occurring (e.g., the presence of a botanical taxa).
- *β*0 is the intercept (constant term).
- *βi* are the coefficients for each explanatory variable.
- *Xi* are the explanatory variables (altitude, temperature, precipitation, age range, and data source).

Based on our variables, the model equation can be written as follows:

Logit(*p*) = *β*0 + *β*1×Altitude1 + *β*2 × Altitude2 + *β*3 × Altitude3 + *β*4 × Temperature1 + *β*5 × Temperature2 + *β*6 × Precipitation1 + *β*7 × Precipitation2 + *β*8 × Age1 + *β*9 × Age2 + *β*10 × Data Source1 + *β*11 × Data Source2

where

- Altitude1, Altitude2, and Altitude are the dummy variables for the three levels of Altitude (600–1600 m, 1600–2400 m, and 2400–3031 m).
- Temperatures 1 and 2 represent the dummy variables for the two levels of temperature average (5 to 12 °C and 7 to 13 °C).
- Precipitations 1 and 2 represent the dummy variables for the two levels of Precipitation average (1400–1600 mm and 1200–1400 mm).
- Age1 and Age2 are the dummy variables for the two levels of Age Range (71–75 years and 30–80 years).
- Data Source has two levels: Interviews and Herbarium, with coefficients substituted accordingly.

These analyses will help reveal how both ecological changes and cultural transformations contribute to the erosion or revitalization of TEK in the region.

The collected data will also be examined through frequency analysis to track patterns in plant species use across different altitudinal zones and over time, highlighting how knowledge is retained, lost, or adapted in response to changing circumstances. Additionally, Venn diagrams will be used to represent the overlap between plant species used in various cultural contexts and ecological zones, offering a visual representation of shared or distinct knowledge systems.

## 5. Conclusions

In conclusion, the significant decline in the use of herbs in the Alpine Southern Occitan region reflects a broader erosion of traditional ecological knowledge, primarily due to diminished daily interactions with nature. The application of the Hysteresis Effect theory emphasizes the challenges of reversing this decline. Yet, it also offers hope for recovery by revitalizing uses of plants such as *Achillea, Artemisia, Veronica*, and *Viola* spp. Although these plants remain in the landscape, their cultural and practical significance has diminished; to reintroduce them into contemporary food and beverage markets could revamp both interest in and knowledge of these plants, paving the way for a more sustainable and bioculturally informed future.

## Figures and Tables

**Table 1:** Plant species used for food and medicinal purposes in the Maira, Grana, and Stura Valleys ((F): food; (FM): food and medicinal; and (M): medicinal) and (p: present; a: absent).

Botanical Taxa	Family	Rovera (1982) [22]	Musset and Dore (2004) [23]	Our Collected Data (2011)	Our Collected Data 2022	Part Used	Usage	Methods of Preparations and Usage	Data Reference
*Abies alba* Mill.	Pinaceae	p	p	a	a	Wood	F	Timber, ornamental purposes	Musset and Dore [23]
		a	a	a	a	Sap	M	The sap can be used in tinctures or syrups for respiratory issues or as a topical antiseptic	Rovera et al. [22]
*Abies grandis* (Douglas ex D.Don) Lindl.	Pinaceae	a	p	a	a	Wood	F	Timber, ornamental purposes	Musset and Dore [23]
*Abies nordmanniana* (Steven) Spach	Pinaceae	a	p	a	a	Wood	F	Timber, ornamental purposes	Musset and Dore [23]
*Acacia* spp.	Fabaceae	a	a	a	p	Flowers, bark	F	Used in fritters, omelets, and as flavoring. Only white or pink flowers used	Data (2022)
*Achillea erba-rotta* All.	Asteraceae	p	a	a	a	Aerial part	M	Decoction or tea. Drink 1–2 cups per day	Rovera et al. [22]
*Achillea millefolium* L.	Asteraceae	p	p	p	a	Flowers, leaves	M	Herbal remedy, anti-inflammatory	Musset and Dore [23]
*Aconitum napellus* L.	Ranunculaceae	p	p	a	a	Roots, leaves	M	Medicinal uses, toxicity	Musset and Dore [23]
*Adiantum capillus-veneris* L.	Pteridaceae	a	p	a	a	Leaves	M	Treats respiratory issues	Musset and Dore [23]
*Aesculus hippocastanum* L.	Sapindaceae	a	p	a	a	Seeds	M	Medicinal (for circulation)	Musset and Dore [23]
*Agrimonia eupatoria* L.	Rosaceae	a	a	p	a	Leaves	F	Herbal teas and infusions	Data (2011)
*Alliaria petiolata* (M.Bieb.) Cavara and Grande	Brassicaceae	a	a	a	p	Leaves, flowers, roots	FM	Used as broccoletti or in pasta Seeds used to make a mustard-like sauce	Data (2022)
*Allium cepa* L.	Amaryllidaceae	p	a	a	a	Bulb	FM	Raw or cooked as food; used in folk medicine for colds, coughs, and as an antibacterial	Rovera et al. [22]
*Allium porrum* L.	Amaryllidaceae	p	a	a	a	Leaves, bulbs	FM	Consumed as a vegetable in cooking; also used in herbal teas for its medicinal properties	Rovera et al. [22]
*Allium sativum* L.	Amaryllidaceae	p	a	a	a	Bulb	FM	Eaten raw or cooked, or used in oils or tinctures for its antibacterial, antiviral, and cardiovascular benefits	Rovera et al. [22]
*Allium schoenoprasum* L.	Alliaceae	a	a	p	a	Leaves	F	Food for cows, milk becomes more bitter	Data (2011)
*Allium ursinum* L.	Liliaceae	a	a	p	p	Leaves, bulbs	FM	Used for flavoring vegetables, salads, or dishes with fish Flowers also used in dishes.Treats insomnia, respiratory and cardiac disorders	Data (2011)
*Alopecurus pratensis* L.	Poaceae	a	a	p	a	Leaves and flowers	F	Used to make better cheese	Data (2011)
*Anemone vulgaris* Miller	Ranunculaceae	p	a	a	a	Aerial parts	M	Decoction, drink 1 small glass before meals	Rovera et al. [22]
*Angelica archangelica* L.	Apiaceae	p	a	a	a	Roots, leaves	M	Used in tinctures or teas to treat digestive issues, respiratory conditions, and as a mild sedative.	Rovera et al. [22]
*Angelica sylvestris* L.	Apiaceae	a	p	a	a	Roots, leaves	M	Herbal remedy, digestive aid	Musset [23]
*Antennaria dioica* (L.) Gaertner	Asteraceae	p	a	a	a	Herb	M	Made into an infusion or poultice for treating wounds or as a diuretic	Rovera et al. [22]
*Apium graveolens* L.	Apiaceae	p	p	a	a	Leaves, stems	FM	Used in cooking soups, or salads and herbal medicine for digestive health and as a mild sedative	Musset and Dore [23]
*Arctium lappa* L.	Asteraceae	p	p	p	a	Roots, seeds	M	Herbal remedy for skin, detoxification; root is used in decoctions or teas for detoxification, skin health, and as an anti-inflammatory	Musset and Dore [23]
*Arctostaphylos uva-ursi* (L.) Spreng.	Ericaceae	a	p	a	a	Leaves	M	Urinary health, antiseptic	Musset and Dore [23]
*Armoracia rusticana* G.Gaertn., B.Mey. and Scherb.	Brassicaceae	a	a	p	a	Leaves	F	Liqueur	Data (2011)
*Arnica montana* L.	Asteraceae	p	a	a	a	Flowers, roots	M	Applied topically in ointments or tinctures for bruises, sprains, and inflammatory pain	Rovera et al. [22]
*Artemisia absinthium* L.	Asteraceae	p	p	p	a	Leaves, flowers	FM	Alcoholic beverage, or teas to treat digestive issuesappetite stimulation, and parasitic infections	Musset and Dore [23]
*Artemisia genipi* Stechm.	Asteraceae	p	p	a	a	Flowers, leaves	F	Liqueur production	Musset and Dore [23]
		a	a	a	a	Leaves, flowers	M	Used in herbal liqueurs or teas for digestive support, appetite regulation, and as a stimulant	Rovera et al. [22]
*Artemisia glacialis* L.	Asteraceae	a	a	p	a	Flowers and stems	F	Liqueur	Data (2011)
*Artemisia umbelliformis* Lam.	Asteraceae	p	a	a	a	Aerial parts	M	Infusion: A pinch of plant per cup of water. Drink during the day, avoid overuse	Rovera et al. [22]
*Artemisia vulgaris* L.	Asteraceae	a	p	a	a	Leaves, flowers	FM	Digestive aid, medicinal herb	Musset and Dore [23]
*Aruncus dioicus* (Walter) Fernald	Rosaceae	a	a	p	a	Shoots	F	Sprouts preserved in oil or in omelets	Data (2011)
*Asparagus acutifolius* L.	Liliaceae	a	a	p	a	Leaves and stem	F	Boiled and eaten in salad	Data (2011)
*Atropa belladonna* L.	Solanaceae	a	p	a	a	Roots, leaves, berries	M	Historical medicinal use (toxic)	Musset and Dore [23]
*Barbarea vulgaris* W.T.Aiton	Brassicaceae	a	a	p	a	Leaves	M	Used as a diuretic	Data (2011)
*Borago officinalis* L.	Boraginaceae	a	a	p	a	Flowers	F	Cooked and used in omelets	Data (2011)
*Brassica oleracea* L.	Brassicaceae	p	a	a	a	Leaves	M	Heated leaves with an iron or in the oven, then applied to the affected area. Apply 2–3 times a day	Rovera et al. [22]
*Bunium bulbocastanum* L.	Apiaceae	a	a	p	a	Bulb	F	Used as a substitute for potatoes with milk (or cream) and flour to make cakes, then baked in the oven. Or roasted on a hot stone. Also dried for the winter	Data (2011)
*Clinopodium nepeta* (L.) Kuntze	Lamiaceae	p	a	a	a	Whole plant (flowering)	M	Infusion: A pinch of dried leaves per cup of water. Use compresses as needed	Rovera et al. [22]
*Calendula arvensis* L.	Asteraceae	a	p	a	a	Flowers	FM	Medicinal uses, skin care	Musset and Dore [23]
*Calendula officinalis* L.	Asteraceae	p	p	p	a		M	Skin care, anti-inflammatory	Musset and Dore [23]
		a	a	a	a	Flowers	F	Soups and medicinal uses as an emollient	Data (2011)
		a	a	a	a		M	Infusion: 1–2 flowers in 1 L of water. Apply as a compress or wash	Rovera et al. [22]
*Campanula rapunculus* L.	Campanulaceae	a	a	p	a	Leaves and flowers	F	A liqueur called “Sanvoran” is made from it, typical of the Occitan region	Data (2011)
*Capsella bursa*-*pastoris* Medik.	Brassicaceae	a	a	p	a	Leaves	F	Salads	Data (2011)
*Carlina acaulis* L.	Asteraceae	a	p	a	a	Roots	M	Medicinal purposes	Musset and Dore [23]
*Carlina vulgaris* L.	Asteraceae	p	a	a	a	Aerial parts	M	Infusion: 1 tablespoon per cup of water. Drink after meals	Rovera et al. [22]
*Carum carvi* L.	Apiaceae	a	p	a	a	Seeds	FM	Culinary uses, digestive aid	Musset and Dore [23]
*Castanea sativa* Mill.	Fagaceae	a	p	p	a	Nuts, wood, fruits	F	Edible nuts, timber, roasted or boiled, sweet or salty	Musset and Dore [23]
*Celtis australis* L.	Ulmaceae	a	a	p	a	Seeds	F	Oil	Data (2011)
*Centaurea cyanus* L.	Asteraceae	p	a	a	a	Flowers	M	Infusion: Flowers in water. Use as an eyewash or compress	Rovera et al. [22]
*Cetraria islandica* (L.) Ach.	Caryophyllaceae	p	a	a	a	Thallus, Lichen (tallo)	M	Decoction, drink 1 glass per day	Rovera et al. [22]
*Chelidonium majus* L.	Papaveraceae	p	a	a	a	Latex, root	M	Apply latex topically to affected areas or use decoction of root (10 cm in 1 L of water). Drink a small cup before meals	Rovera et al. [22]
*Chenopodium bonus-*henricus L.	Amaranthaceae	p	a	p	p	Leaves, stems	FM	Often boiled and mixed with other vegetables. Used in a casserole with Melissa. Cooked in agnolotti, raw in gnocchi. Grows well on slopes.	Stellato (2022)
*Chrysojasminum odoratissimum* (L.) Banfi	Oleaceae	p	a	a	a	Leaves	M	Decoction: 4–5 leaves in 2 L of water for 30 min	Rovera et al. [22]
*Cicerbita alpina* Wallr.	Asteraceae	a	a	p	a	Leaves	F	Used in salads	Data (2011)
*Cichorium intybus* L.	Asteraceae	p	a	p	p	Roots, leaves	F	Poor man’s coffee, used as an antidote against worms, also in salads	Data (2011)
*Cinchona calisaya* Wedd.	Rubiaceae	p	a	a	a	Root	M	Decoction, drink 1 small glass after meals	Rovera et al. [22]
*Cinnamomum verum* J.Presl	Lauraceae	p	a	a	a	Bark	M	Infusion: 1 L of water, 1 tsp thyme, 2 of burdock root, left overnight. Drink 1 cup after every meal	Rovera et al. [22]
*Citrus limon* (L.) Osbeck	Rutaceae	p	a	a	a	Fruit	FM	Fresh juice, drink the juice of 1/2 lemon daily	Rovera et al. [22]
*Cornus sanguinea* L.	Cornaceae	a	a	p	a	Seeds	F	Oil	Data (2011)
*Corylus avellana* L.	Betulaceae	a	a	p	a	Fruits	F	Oil	Data (2011)
*Crataegus monogyna* Jacq.	Rosaceae	p	a	a	a	Flower buds with leaves	M	Decoction. Drink after meals	Rovera et al. [22]
*Cynodon dactylon* (L.) Pers.	Poaceae	p	a	a	a	Entire plant	M	Decoction or infusion. Drink after meals or as needed	Rovera et al. [22]
*Diplotaxis tenuifolia* (L.) DC.	Brassicaceae	a	a	p	p	Leaves	F	Used raw and cooked in meats, fish, and cheeses. Flower buds used in pasta sauce with anchovies	Data (2011)
*Dryopteris filix-mas* (L.) Schott	Dryopteridaceae	a	p	a	a	Rhizomes, leaves	M	Traditionally used in herbal remedies	Musset and Dore [23]
*Echium vulgare* L.	Boraginaceae	p	a	a	a	Flowers	M	Decoction, drink 1 glass per day	Rovera et al. [22]
*Equisetum arvense* L.	Equisetaceae	a	a	p	a	Stem	F	Salads	Data (2011)
*Equisetum* spp.	Equisetaceae	p	a	a	a	Aerial parts	M	Decoction, drink 1 glass per day	Rovera et al. [22]
*Festuca rubra* L.	Poaceaea	a	a	p	a	Leaves and flowers	F	Used to improve cheese quality	Data (2011)
*Foeniculum vulgare* Mill.	Apiaceae	a	a	p	a	Leaves	F	Used to flavor dishes and drinks, or also to make liqueurs.	Data (2011)
*Fragaria vesca* L.	Rosaceae	p	a	a	a	Leaves	M	Infusion: A handful of dried leaves in 1/2 L of water	Rovera et al. [22]
*Fraxinus excelsior* L.	Oleaceae	p	a	p	a	Leaves	M	Leaves used as diuretics and sudorifics	Data (2011)
*Fumana ericoides (*Cav.) Gand.	Cistaceae	p	a	a	a	Flowers	M	Infusion: 5–6 flowers in 1/2 L of water	Rovera et al. [22]
*Galium album* Mill.	Rubiaceae	p	a	a	a	Flowers	M	Infusion: A pinch of flowers in water	Rovera et al. [22]
*Gentiana acaulis* L.	Gentianaceae	p	a	a	a	Flowers	M	Maceration: 20 flowers in 1 L of red wine for 10 days	Rovera et al. [22]
*Gentiana lutea* L.	Genzianaceae	p	a	p	p	Flowers, roots	F	Root used after being washed, cut, and dried, commonly used in liqueurs and aromatic wines.Food for cows, milk becomes more bitter, but it is also used for making liqueurs	Data (2011)
		a	a	a	a		M	Decoction: 1.5 L of water and 15 pieces of root (4–5 cm)	Rovera et al. [22]
*Gentiana acaulis* L.	Genzianaceae	a	a	p	a	Flowers	F	Food for cows, milk becomes more bitter, also used in liqueurs	Data (2011)
*Glycyrrhiza glabra* L.	Fabaceae	a	p	a	a	Roots	FM	Used as a sweetener and in herbal medicine	Musset and Dore [23]
*Hedera helix* L.	Araliaceae	p	a	a	a	Leaves	M	Decoction: 10–15 leaves in 1 L of water	Rovera et al. [22]
*Helianthus* spp.	Asteraceae	a	a	p	a	Tuber	F	Eaten raw	Data (2011)
*Hylotelephium telephium* (L.) H.Ohba	Crassulaceae	p	a	a	a	Aerial parts (Flowers)	M	Infusion: 1 tablespoon dried plant in ½ liter of water	Rovera et al. [22]
*Humulus lupulus* L.	Cannabaceae	a	a	p	a	Leaves, flowers	F	Digestive liqueurs made from the flowers, the sprouts are used in soups, omelets, and as a side dish for polenta.	Data (2011)
*Hypericum perforatum* L.	Hypericaceae	p	a	a	a	Whole plant	M	For colds, apply to the burned area several times a day	Rovera et al. [22]
*Hyssopus officinalis* L.	Asteraceae	a	a	p	a	Leaves	M	Perfumes and medicines for the lungs are made from it	Data (2011)
*Juglans regia* L.	Juglandaceae	p	a	p	a	Fruit	F	Oil	Data (2011)
		a	a	a	a	Leaves	M	Decoction: 2–3 handfuls of leaves in 5–6 L of water	Rovera et al. [22]
*Juniperus communis* L.	Cupressaceae	p	a	p	p	Berries, roots	F	Used in cheese refining, and roots for liqueur production	Data (2011)
		a	a	a	a		F	Berries used in meats, game, pork, rabbit, vegetables, pickled mushrooms. Used in liquor making, especially gin	Stellato (2022)
		a	a	a	a		FM	Decoction (5–7 berries), soaking in wine or water, or consumed raw after meals	Rovera et al. [22]
*Laburnum anagyroides* Medik.	Fabaceae	p	a	a	a	Bark, young branches	M	Decoction: 50 cm of dry bark in 1 L of water; young branches ground with vinegar for poultices	Rovera et al. [22]
*Lactuca perennis* L.	Asteraceae	a	p	a	a	Leaves	FM	Edible, medicinal uses	Musset and Dore [23]
*Lactuca serriola* L.	Asteraceae	a	a	p	a	Leaves	F	Salads and soups. Used as a laxative	Data (2011)
*Lactuca virosa* Thunb.	Asteraceae	a	a	a	p	Leaves, stem	F	Tender leaves used in salads Rosettes used in creams, soups, and mashed potatoes	Stellato (2022)
*Lamium album* L.	Lamiaceae	p	a	p	a	Leaves	M	Decoction or used for inflammation in the genital tract	Rovera et al. [22]
*Lamium purpureum* L.	Lamiaceae	p	a	a	a	Aerial parts	M	Used externally for treating wounds and inflammations	Rovera et al. [22]
*Lapsana communis* L.	Asteraceae	a	a	p	a	Leaves	F	Soups and omelets	Data (2011)
*Larix decidua* (L.) Mill.	Pinaceae	p	p	a	a	Wood, resin	FM	Timber, ornamental. Applied to abscesses to promote maturation	Rovera et al. [22]
*Lathyrus oleraceus* Lam.	Fabaceae	a	a	p	a	Fruits	F	Edible but also a bit poisonous	Data (2011)
*Lathyrus sativus* L.	Fabaceae	p	a	a	a	Dry plant (flowering)	M	Secondary use to expel the placenta	Rovera et al. [22]
*Lathyrus tuberosus* L.	Fabaceaea	a	a	p	a	Tubers and leaves	F	Leaves in salads. Tubers in soups or salads once cooked. It was also called “hunger herb” because it was used during times of extreme famine. Otherwise, it was eaten only by cows	Data (2011)
*Laurus nobilis* L.	Lauraceae	a	p	a	a	Leaves	FM	Culinary uses, anti-inflammatory	Musset and Dore [23]
*Lavandula angustifolia* Mill.	Lamiaceae	p	p	a	a	Flowers, leaves	M	Aromatherapy, skin care	Musset and Dore [23]
*Lavandula stoechas* L.	Lamiaceae	a	a	p	a	Flowers	F	Ornamental, honey	Data (2011)
*Leontopodium nivale* subsp. *alpinum* (Cass.) Greuter	Asteraceae	p	a	a	a	Whole plant	M	Decoction: 3–4 flowers in 1 L of water	Rovera et al. [22]
*Levisticum officinale* W.D.J.Koch	Apiaceae	a	a	p	a	Stems and leaves	F	In summer, only the leaves are used, while, in spring, the stem is also used. Used chopped on Castelmagno cheese cubes	Data (2011)
*Lilium martagon* var*. martagon*	Liliaceae	a	a	p	a	Bulb	F	Salads	Data (2011)
*Linum usitatissimum* L.	Linaceae	a	p	a	a	Seeds, fiber	F	Fiber production, oil extraction	Musset [23]
*Lupinus angustifolius* L.	Fabaceaea	a	a	p	a	Fruits	F	Used as a substitute for fava beans, after being thoroughly washed to remove toxic substances.	Data (2011)
*Lythrum salicaria* L.	Lythraceae	a	p	a	a	Flowers, roots	M	Ornamental, urinary health	Musset and Dore [23]
*Malva alcea* L.	Malvaceae	p	a	a	a	Inflorescences, roots	M	Decoction: 1 handful in 1 L of water; compresses applied to the legs	Rovera et al. [22]
*Malva pusilla* Sm.	Malvaceae	p	a	a	a	Aerial parts, flowers	M	Decoction or infusion: 5–6 flowers in water or 1 plant in 2–3 L	Rovera et al. [22]
*Malva sylvestris* L.	Malvaceae	p	p	p	p	Leaves, flowers	FM	Soothing digestive, skin care	Musset and Dore [23]
		a	a	a	a	Leaves, flowers, and roots	FM	Raw in salads, cooked as an antispasmodic for the intestines. Roots against indigestion. Also used as a refreshing agent. Once boiled, it was used for inflammations	Data (2011)
		a	a	a	a	Leaves, flowers	FM	Paired with herbs for fillings or omelets. Buds pickled in vinegar as a condiment.Used for cough, bronchitis, and digestive issues. In the past, used in soups for children or elderly with stomach or bronchitis issues	Stellato (2022)
		a	a	a	a	Entire plant, leaves	M	Decoction: Used for inflammation, gargles, or anti-inflammatory purposes	Rovera et al. [22]
*Marrubium vulgare* L.	Lamiaceae	p	p	a	a	Leaves, flowers	M	Respiratory health, cough remedy	Musset [23]
		a	a	a	a	Entire plant	M	Decoction: 1 plant in 5 cups of water	Rovera et al. [22]
*Matricaria chamomilla* L.	Asteraceae	p	p	a	a	Flowers	M	Known for its calming properties	Musset and Dore [23]; Rovera et al. [22]
*Matricaria recutita L.*	Asteraceae	a	a	p	a	Flowers	FM	Infusions	Data (2011)
*Melilotus officinalis* (L.) Lam.	Fabaceae	p	p	a	a	Leaves	M	Infusion: 3–4 leaves in 1 L of water	Rovera et al. [22]
		a	a	a	a	Flowers, leaves	M	Blood circulation, agricultural use	Musset and Dore [23]
*Melissa officinalis* L.	Lamiaceae	a	p	p	p	Leaves	FM	Calming, digestive aid	Musset and Dore [23]
		a	a	a	a		F	Used to give the characteristic flavor in salads	Data (2011)
		a	a	a	a		FM	Used raw in salads, soups, omelets. Commonly used in liquors and as an aromatic ingredient. Used for depression, kidney colic, insomnia, and insect bites	Stellato (2022)
*Mentha × rotundifolia* (L.) Huds.	Labiateae	a	a	p	a	Leaves	F	Used to flavor dishes and drinks	Data (2011)
*Mentha aquatica* L.	Lamiaceae	p	a	a	a	Leaves	FM	Infusion: 3–4 leaves per cup of water	Rovera et al. [22]
*Mentha piperita* L.	Lamiaceae	p	a	a	a	Leaves	FM	Decoction or infusion, used for digestion and colic relief	Rovera et al. [22]
*Mentha* sp.	Lamiaceae	a	p	a	a	Leaves, flowers	FM	Digestive aid, culinary uses	Musset and Dore [23]
*Mespilus germanica* (L.) Kuntze	Rosaceae	a	p	a	a	Fruit	F	Edible fruit, ornamental	Musset and Dore [23]
*Muscari botryoides* (L.) Mill.	Liliaceae	a	a	p	a	Bulb	F	The bulb is roasted and dried for the winter	Data (2011)
*Myosotis* spp.	Boraginaceae	a	p	a	a	Flowers	F	Symbolic uses, ornamental	Musset and Dore [23]
*Nasturtium officinale* R.Br.	Brassicaceae	p	p	p	a	Aerial parts	FM	Likely consumed raw or prepared as an infusion for diuretic or digestive benefits	Rovera et al. [22]
		a	a	a	a	Leaves, stems	FM	Culinary uses, detoxification	Musset and Dore [23]
		a	a	a	a	Leaves	M	Salads, decoctions, hair growth	Data (2011)
*Nepeta cataria* L.	Lamiaceae	a	p	a	a	Leaves, flowers	M	Cat attraction, medicinal uses	Musset and Dore [23]
*Ocimum basilicum* L.	Lamiaceae	a	p	a	a	Leaves	FM	Culinary uses, digestive aid	Musset and Dore [23]
*Olea europaea* L.	Oleaceae	p	p	a	a	Fruit	F	Olive oil production, culinary uses	Musset and Dore [23]
		a	a	a	a	Leaves, oil	M	Used for treating burns, likely as oil or leaf extracts	Rovera et al. [22]
*Onopordon acanthium* L.	Asteraceae	a	a	p	a	Seeds	F	Oil	Data (2011)
*Origanum vulgare* L.	Lamiaceae	p	p	p	a	Leaves, flowers	FM	Culinary uses, medicinal uses	Musset and Dore [23]
		a	a	a	a	Leaves	F	Used to flavor dishes and drinks	Data (2011)
		a	a	a	a	Flowers	M	Decoction, 2–3 times a day for knee application	Rovera et al. [22]
*Oxalis acetosella* L.	Oxalidaceae	a	a	a	p	Leaves, flowers	M	Leaves and stems used in soups, roasts, or to make a lemonade-like drink. Astringent, diuretic, blood purifier. Used for gastric issues, liver congestion, nephritis, skin rashes, and worms	Stellato (2022)
*Papaver rhoeas* L.	Papaveraceae	a	p	p	a	Flowers	M	Soothing, medicinal	Musset and Dore [23]
		a	a	a	a	Leaves	F	Baked to make green pies. Or in salads	Data (2011)
*Parietaria judaica* L.	Urticaceae	a	p	a	a	Leaves, stems	M	Respiratory health, herbal remedy	Musset and Dore [23]
*Parietaria officinalis* L.	Urticaceae	p	a	p	a	Leaves	M	Poultice of chopped leaves; infusion with a handful of leaves in 1 L of water	Rovera et al. [22]
		a	a	a	a	Leaves and bulb	FM	Salads, soups, omelets. The juice was used as a diuretic and detoxifier for the urinary tract. Bulbs were eaten after being boiled twice to remove the bitter taste, then fried in slices or roasted	Data (2011)
*Pastinaca sativa* L.	Apiaceae	a	p	a	a	Roots	FM	Culinary uses, medicinal uses	Musset and Dore [23]
*Petroselinum crispum* (Mill.) Fuss	Apiaceae	p	p	a	a	Aerial parts	F	Infusion: 2 umbels in one cup of water	Rovera et al. [22]
		a	a	a	a	Leaves	FM	Culinary uses, digestive aid	Musset and Dore [23]
*Peucedanum ostruthium* W.D.J.Koch	Apiaceae	p	p	a	a	Roots	M	Roots must be crushed and prepared as a decoction	Rovera et al. [22]; Musset and Dore [23]
*Phyteuma orbiculare* L.	Campanulaceae	a	a	p	a	Leaves, inflorescences, and roots	F	Cooked and then used in omelets, roots are consumed in salads	Data (2011)
*Phyteuma ovatum* Honck.	Campanulaceae	a	a	p	a	Leaves, inflorescences, and roots	F	Oil is made from it, or it is eaten toasted	Data (2011)
*Pimpinella anisum* L.	Apiaceae	p	p	p	p	Seeds, leaves	FM	Used for flavoring and medicinal purposes	Musset and Dore [23]
		a	a	a	a	Leaves	M	Eaten with snails. The flowers are rarely used because they are laxative	Data (2011)
		a	a	a	a	Umbels	FM	Infusion: 2 leaves or umbels in a cup of water	Rovera et al. [22]
		a	a	a	a	Seeds, leaves	F	Fresh leaves used in soups, cheeses, and cooked vegetables	Stellato (2022)
*Pinus cembra* L.	Pinaceae	a	p	p	a	Timber, nuts	F	Used for timber and nuts	Musset and Dore [23]
		a	a	a	a	Seeds	F	Salads with the leaves and dried rhizome as a digestive	Data (2011)
*Pinus sylvestris* L.	Pinaceae	a	p	a	a	Timber, resin	F	Used for timber and resin	Musset and Dore [23]
*Plantago lanceolata* L.	Plantaginaceae	p	a	a	a	Leaves	M	Decoction or poultice for wounds and respiratory relief	Rovera et al. [22]
*Plantago major* L.	Plantaginaceae	a	p	a	a	Leaves, seeds	M	Common herb for medicinal uses	Musset and Dore [23]
		a	a	a	a	Basal leaves	M	Decoction: 2–3 roots in one cup of water; one cup in the evening	Rovera et al. [22]
*Plantago* sp.	Plantaginaceae	a	a	p	a	Leaves	M	Used against pimples	Data (2011)
*Poa pratensis* L.	Poaceaea	a	a	p	a	Flowers and leaves	F	Used to make better cheese	Data (2011)
*Polygala* spp.	Polygalaceae	a	p	a	a	Roots, leaves	M	Used in traditional medicine	Musset and Dore [23]
*Polygonum bistorta* Samp.	Poligonaceae	a	a	p	a	Leaves	M	The leaves are used to make a powerful medicine for hemorrhoids	Data (2011)
*Polypodium vulgare* L.	Polypodiaceae	p	a	a	a	Root	M	Decoction: a handful of root in 1 L of water; drink several times during the day	Rovera et al. [22]
*Polyporus officinalis* (Vill.) Fr.	Polyporaceae	p	a	a	a	Fungi	M	Decoction: drink 1–2 cups per day	Rovera et al. [22]
*Portulaca oleracea* L.	Portulacaceae	a	p	a	a	Leaves, seeds	FM	Edible herb used in salads and for medicinal properties	Musset and Dore [23]
*Primula veris* L.	Primulaceae	p	a	p	a	Buds and leaves	FM	Used in potato flan, soups with other herbs, or in omelets. Also used as diuretics and detoxifiers. Buds pickled or with sugar	Data (2011)
		a	a	a	a	Flowers and leaves	M	Decoction: use flowers and leaves	Rovera et al. [22]
*Primula vulgaris* Huds.	Primulaceae	a	p	a	a	Flowers, leaves	FM	Used ornamentally and for medicinal teas	Musset and Dore [23]
*Prunus avium* (L.) L.	Rosaceae	p	p	a	a	Fruit	F	Produces edible fruit	Musset and Dore [23]
		a	a	a	a	Stems	M	-	Rovera et al. [22]
*Prunus cerasus* L.	Rosaceae	p	a	a	a	Stems	M	-	Rovera et al. [22]
*Prunus spinosa* L.	Rosaceae	a	p	a	a	Berries	FM	Used in jams and liqueurs	Musset and Dore [23]
*Pulmonaria officinalis* L.	Boraginaceae	p	a	a	p	Leaves, flowers	FM	Leaves used in fried dishes, fillings, pies, ravioli. Emollient, rich in vitamins A and C	Stellato (2022)
		a	a	a	a	Leaves	M	-	Rovera et al. [22]
*Quercus robur* L.	Fagaceae	p	a	a	a	Bark	M	Decoction, drink 1 small glass after meals	Rovera et al. [22]
*Ranunculus acris* L.	Ranunculaceae	p	p	a	a	Flowers, Leaves	M	Toxic plant often found in meadows	Musset and Dore [23]
		a	a	a	a	Bulb (sliced)	M	Decoction: 5–6 fruits in 4 L of water; decoction 4–5 times a day	Rovera et al. [22]
*Rheum rhabarbarum* L.	Polygonaceae	a	p	a	a	Stems, roots	F	Used in cooking and desserts	Musset and Dore [23]
*Ribes rubrum* L.	Grossulariaceae	a	p	a	a	Fruit	F	Used in jams and desserts	Musset and Dore [23]
*Rorippa* spp.	Brassicaceae	a	p	a	a	Leaves, stems	FM	Culinary uses, medicinal uses	Musset and Dore [23]
*Rosa canina* L.	Rosaceae	p	p	a	a	Fruit, flowers	FM	Used for medicinal purposes and in jams	Musset and Dore [23]
		a	a	a	a	Fruit	M	Decoction: 5–6 leaves per cup of water; Drink 2–3 times a day	Rovera et al. [22]
*Rosa canina* L.	Rosaceae	a	a	p	a	Fruits	F	Used to make sauces. Or toasted as a tea substitute	Data (2011)
*Rosa moschata* Herrm.	Rosaceae	a	p	a	a	Flowers	F	Known for its fragrant flowers	Musset and Dore [23]
*Rosmarinus officinalis* L.	Lamiaceae	p	p	a	a	Leaves, flowers	FM	Fragrant herb used in cooking and medicine	Musset and Dore [23]
		a	a	a	a	Leaves	M	Decoction: 7–8 cm of twigs in 1 L of water; drink 3 times a day	Rovera et al. [22]
*Rubus fruticosus* L.	Rosaceae	p	p	a	a	Fruit, leaves	F	Known for its berries (blackberries)	Musset and Dore [23]
		a	a	a	a	Leaves	M	Decoction: 2–3 leaves per cup of water; drink 3 times a day	Rovera et al. [22]
*Rubus idaeus* L.	Rosaceae	a	p	a	a	Fruit	F	Edible fruit commonly used in jams and desserts	Musset and Dore [23]
*Rumex acetosa* L.	Polygonaceae	a	p	p	a	Leaves, roots	FM	A sour leafy plant often used in salads	Musset and Dore [23]
*Rumex alpinus* L.	Polygaceae	a	a	p	a	Rhizome and leaves	F	Baked with or without rice, seasoned with butter, cheese, and eggs to make green pies, a holiday dish	Data (2011)
*Rumex crispus* L.	Polygonaceae	p	a	a	a	Root	M	Decoction: 6–7 cm of root in 3 glasses of water; Drink 1 small glass in the morning	Rovera et al. [22]
*Rumex obtusifolius* L.	Polygonaceae	p	a	a	a	Leaves	M	Decoction: 4–5 leaves in 1 L of water on an empty stomach; Drink 1 glass in the morning on an empty stomach for 15 days	Rovera et al. [22]
*Rumex patientia* L.	Polygonaceae	a	p	a	a	Leaves, roots	M	Wild herb with medicinal properties	Musset and Dore [23]
*Ruta graveolens* L.	Rutaceae	p	a	p	p	Leaves	F	Grappa	Data (2011)
		a	a	a	a	Leaves, flowers	F	Used in salads or with herbs to balance strong flavors. Stems used like broccoli, boiled and seasoned.	Stellato (2022)
		a	a	a	a	Leaves	M	Grappa preparation, drink 1 small glass after meals	Rovera et al. [22]
*Salix alba* L.	Salicaceae	a	p	a	a	Bark	M	Used for its bark’s medicinal properties	Musset and Dore [23]
*Salix* spp.	Salicaceae	p	p	a	a	Leaves	M	Crushed leaves used as toothpaste; apply 2 times a day	Rovera et al. [22]
		a	a	a	a	Bark, leaves	M	Known for its use in herbal medicine	Musset and Dore [23]
*Salvia officinalis* L.	Lamiaceae	p	a	a	p	Leaves	M	Decoction: 1⁄2 umbrella in 1⁄2 L of water; drink 1 small cup in the morning	Rovera et al. [22]
		a	a	a	a	Leaves, flowers	FM	Flowers fried in batter, used in sauces or soups. Used in a hot drink with lemon for digestion.	Stellato (2022)
*Salvia pratensis* L.	Lamiaceae	a	a	p	a	Leaves	F	Omelets, salads, soups. Dried flowers used as flour to make bread. Also animal feed	Data (2011)
*Sambucus nigra* L.	Adoxaceae	p	p	p	a	Berries, flowers	M	Immune boosting, cold remedy	Musset and Dore [23]
		a	a	a	a	Leaves and flowers	FM	Soups, salads, omelets. Preparation of elderberry wine. Jam is made, which has a laxative effect. Flowers are fried in batter. A liqueur is also made	Data (2011)
		a	a	a	a	Fruits	M	Wine made by pressing berries; Vulnerary (wound healing)	Rovera et al. [22]
*Sanguisorba minor* Scop.	Rosaceae	p	a	a	a	Flowers	M	Decoction: a handful of flowers in 1 L of water	Rovera et al. [22]
*Santolina chamaecyparissus* L.	Asteraceae	a	p	a	a	Leaves, flowers	FM	Known for its aromatic leaves used in herbal remedies	Musset and Dore [23]
*Saponaria officinalis* L.	Caryophyllaceae	a	p	a	a	Roots, leaves	M	Used traditionally to make soap	Musset and Dore [23]
*Satureja hortensis* L.	Lamiaceae	a	p	a	a	Leaves	F	A culinary herb used for flavoring dishes	Musset and Dore [23]
*Satureja montana* L.	Labiateae	p	a	p	p	Leaves	F	Adds flavor to food.	Data (2011)
		a	a	a	a	Aerial parts (flowers)	M	Infusion: 1 tablespoon of dried plant in ½ liter of water	Rovera et al. [22]
		a	a	a	a	Leaves, flowers	F	Used with eggs, legumes, vegetables. Often added to minestrone or savory pudding in Piedmont	Stellato (2022)
*Silene vulgaris* (Moench) Garcke	Cariofillaceae	a	a	p	a	Flowers and flowers	FM	Liqueurs, soups from cooked flowers, and green omelets baked in the oven	Data (2011)
*Silybum marianum* (L.) Gaertn.	Asteraceae	a	a	p	p	Flowers and fruits	F	Cooked leaves used as a liver detoxifier	Data (2011)
		a	a	a	a	Seeds, leaves, flowers	FM	Tender central shoots used raw in salads. Flower receptacles can be boiled or used like artichokes	Stellato (2022)
*Solanum dulcamara* L.	Solanaceae	p	p	a	a	Stems, leaves	M	Known for its toxic and medicinal uses	Musset [23]
		a	a	a	a	Stem	M	Decoction: 10 cm of stem in a cup of water	Rovera et al. [22]
*Solanum tuberosum* L.	Solanaceae	p	a	a	a	Tuber	F	Infusion: 1 leaf per cup of water	Rovera et al. [22]
*Sorbus aucuparia* L.	Rosaceae	a	p	a	a	Berries, leaves	FM	Known for its berries and use in medicinal syrups	Musset and Dore [23]
*Stellaria media* (L.) Vill.	Caryophyllaceae	a	a	p	a	Leaves	F	Liqueur	Data (2011)
*Tanacetum balsamita* L.	Asteraceae	a	a	p	a	Leaves	F	Used in omelets	Data (2011)
*Tanacetum vulgare* L.	Asteraceae	p	p	p	a	Flowers, leaves	M	Known for its medicinal use	Musset and Dore [23]
		a	a	a	a	Leaves and flowers, roots	FM	Salads, condensed, coffee, leaves cooked in butter, soup with herbs, and raw in salad Used against jaundice and gallstones. Buds were pickled and used as capers. Roots toasted as a coffee substitute. A liqueur is also made from the leaves	Data (2011)
		a	a	a	a	Flowers	M	Decoction: 1 L of water, 2–3 flowers of tansy, 1 sprig of wormwood, boiled for 30 min	Rovera et al. [22]
*Taraxacum officinale* F.H.Wigg.	Asteraceae	p	p	p	a	Roots, leaves, flowers	M	Often used in herbal remedies	Musset and Dore [23]
		a	a	a	a	Leaves	FM	Teas, infusions, digestion, gnocchi, cheese refining, and green cakes baked in the oven.	Data (2011)
		a	a	a	a	Leaves	M	Infusion: 1 plant in 1 glass of water	Rovera et al. [22]
*Teucrium chamaedrys* L.	Lamiaceae	p	p	a	a	Leaves, flowers	M	A medicinal plant	Musset [23]
		a	a	a	a	Aerial parts	M	Decoction: 1 glass of water with a pinch of plant	Rovera et al. [22]
*Teucrium montanum* L.	Lamiaceae	a	p	a	a	Leaves, flowers	M	Used for its medicinal qualities	Musset and Dore [23]
*Thymus serpyllum* L.	Lamiaceae	p	p	p	a	Leaves	FM	Salads, teas, and infusions to eliminate intestinal gas and facilitate bile flow	Data (2011)
		a	a	a	a	Leaves, flowers	FM	Used for its aromatic and medicinal properties	Musset and Dore [23]
		a	a	a	a	Aerial parts	M	Decoction: handful in 1 L of water	Rovera et al. [22]
*Thymus vulgaris* L.	Lamiaceae	a	p	a	a	Leaves, flowers	FM	Commonly used in cooking and herbal medicine	Musset and Dore [23]
*Tilia cordata* Mill.	Tiliaceae	p	p	p	a	Flowers, leaves	M	Known for its calming tea	Musset and Dore [23]
		a	a	a	a	Flowers	F	Used to flavor dishes	Data (2011)
		a	a	a	a	Flowers	M	Infusion: 1 teaspoon per cup of water	Rovera et al. [22]
*Tragopogon pratensis* L.	Asteraceae	a	a	p	a	Leaves and roots	FM	Sprouts and leaves used as vegetables, cooked or raw. Especially in soups. Used in green cakes baked in the oven. Roots eaten cooked. Used (unconsciously) against diabetes	Data (2011)
*Trifolium pratense* L.	Fabaceae	a	p	p	a	Flowers, leaves	FM	Used in teas and for its medicinal properties	Musset and Dore [23]
		a	a	a	a	Leaves	F	The bulb is roasted and dried for the winter	Data (2011)
*Tulipa sylvestri* L.	Liliaceae	a	a	p	a	Bulb	F	Paired with roe deer, in sweets, or as a concentrate	Data (2011)
*Tussilago farfara* L.	Asteraceae	p	p	a	a	Leaves, flowers	M	Used for cough and respiratory issues	Musset and Dore [23]
		a	a	a	a	Flowers	M	Infusion: pinch per cup of water	Rovera et al. [22]
*Ulmus minor* Mill.	Ulmaceae	p	a	a	a	Bark	M	Decoction: 4–6 plants in 2 L of water, boil for 4–5 h	Rovera et al. [22]
*Urtica dioica* L.	Urticaceae	p	p	p	p	Leaves, roots	FM	Known for its nutritional and medicinal benefits	Musset and Dore [23]
		a	a	a	a	Fruits	FM	Used in omelets after being well cooked, in soups, or even as shampoo	Data (2011)
		a	a	a	a	Leaves, roots	FM	Used in risotto and ravioli, collected when young and succulent. Diuretic and anti-inflammatory properties	Data (2022)
		a	a	a	a	Whole plant	M	Decoction: handful of leaves in 1% water	Rovera et al. [22]
*Urtica urens* L.	Urticaceae	p	a	a	a	Whole plant	M	Decoction	Rovera et al. [22]
*Urena lobata* subsp. *lobata*	Parmeliaceae	p	a	a	a	Thallus	M	Decoction	Rovera et al. [22]
*Vaccinium myrtillus* L.	Ericaceae	p	p	p	a	Berries, leaves	FM	A plant with medicinal and edible uses	Musset and Dore [23]
		a	a	a	a	Fruit	F	Wine: fruit with abundant sugar, left in the sun or oven	Rovera et al. [22]
		a	a	a	a	Leaves	F	Paired with venison, in desserts or as a concentrate	Data (2011)
*Valerianella locusta* L.	Valerianaceae	a	a	p	a	Leaves	F	Salads	Data (2011)
*Veratrum album* L.	Liliaceae	p	a	a	a	Whole plant	M	Not specified	Rovera et al. [22]
*Verbascum lychnitis* L.	Scrophulariaceae	p	a	a	a	Leaves, seeds and flowers	M	Decoction: one leaf per cup of water	Rovera et al. [22]
*Verbascum thapsus* L.	Scrophulariaceae	p	p	a	a	Flowers, leaves	M	Traditionally used in herbal remedies	Musset and Dore [23]
		a	a	a	a	Flowers	M	Infusion: one teaspoon of dried flowers in a cup of water	Rovera et al. [22]
*Verbena officinalis* L.	Verbenaceae	p	p	a	a	Flowers, leaves	M	Used for its medicinal properties	Musset and Dore [23]
		p	a	p	a	Aerial parts	M	Infusion	Rovera et al. [22]
*Veronica longifolia* subsp*. longifolia*	Scrofulariaceae	a	a	a	a	Leaves	FM	Teas and infusions	Data (2011)
		a	a	a	a	Aerial parts	M	Wine infusion, drink 1 small glass in the morning	Rovera et al. [22]
*Veronica beccabunga* L.	Scrophulariaceae	a	a	p	a	Leaves and flowers	F	Salads	Data (2011)
*Viola alba* Besser	Violaceae	p	a	a	a	Flowers	M	Infusion: 2–3 flowers per cup of water, drink during the headache.	Rovera et al. [22]
*Viola biflora* L.	Violaceae	p	a	a	a	Flowers	M	Infusion: Drink during the headache.	Rovera et al. [22]
*Viola odorata* L.	Violaceae	p	a	a	p	Flowers, leaves	F	Used for decoration, in fritters, and in soups. Use caution as it can cause nausea.	Stellato (2022)
		a	a	a	a	Flowers and leaves	M	Decoction: 5–6 plants in 1 L of water, cook for 2–3 min. Drink after meals for astringent, small cup in the morning on an empty stomach for laxative.	Rovera et al. [22]
*Viola tricolor* L.	Violaceae	p	p	a	a	Flowers	FM	Used for decorative and medicinal purposes	Musset and Dore [23]
		a	a	a	a	Flowers	M	Infusion: 2–3 plants per cup of water. Drink 2–3 small cups during the day	Rovera et al. [22]
*Viscum album* L.	Santalaceae	p	p	a	a	Berries, leaves	M	Used in traditional medicine and rituals	Musset and Dore [23]
		a	a	a	a	Leaves and fruit	M	Infusion: A pinch of flowers per cup of water. Drink 2–3 cups during the day	Rovera et al. [22]
*Vitis vinifera* L.	Vitaceae	p	a	a	a	Fruit	M	Decoction: 7–8 leaves in 1/2 L of water. Drink small cup in the morning	Rovera et al. [22]
*Zea mays* L.	Poaceae	p	a	a	a	Stigmas	M	Decoction: 150 gr. of stigmas in 1 L of water. Drink 3–4 small cups during the day	Rovera et al. [22]

**Table 2:** The logistic regression model was used to analyze the influence of various explanatory variables on the number of botanical taxa observed in different conditions.

Explanatory Variables	Category	Coefficients	Odds Ratios	Std. Error	*p*-Value
Altitude (m)	600–1600	0.8	2.22	0.3	0.002
	1600–2400	0.45	1.57	0.25	0.048
	2400–3031	0.1	1.11	0.32	0.724
Temperature Average (°C)	5 to 12 °C	0.3	1.35	0.28	0.223
	7 to 13 °C	−0.1	0.9	0.27	0.74
Precipitation Average (mm)	1400–1600	−0.2	0.82	0.31	0.511
	1200–1400	−0.5	0.61	0.35	0.151
Age Range (years)	71–75	0.85	2.34	0.3	0.004
	30–80	0.15	1.16	0.22	0.441
Data Source	Interviews	0.6	1.82	0.35	0.09
	Herbarium	0.25	1.28	0.4	0.517

**Table 3:** Ethnobotanical data overview: socio-ecological contexts and methodological approaches across studies and our collected data.

Data	Year	Location	Altitude (m)	Temperature Average (°C)	Precipitation Average (mm)	Age Range	Number of Participants	Data Source	Social and Economic Context
Rovera et al. [22]	1982	Val Maira	600–1600	5 to 10	1300–1500	71–75	Not determined	Direct conversation with locals, isolated area	Isolated economy and social conditions
Musset and Dore [23]	2004	Valle Stura	630–3031	5 to 12	1400–1600	Various (30–80)	24 individuals with diverse professions and roles	Interviews, herbariums, recipe books	Social/economic context needed
Our data collected in 2011	2011	Valle Grana	600–2400	7 to 13	1200–1400	Various (25–75)	20 individuals with diverse professions and roles (e.g., farmers, drivers, herbarium)	Herbarium, Indigenous and Allochthonous Quotes	Multiple generations across various professions (including merchants, restaurateurs, holidaymakers, and others)
Our data collected in 2022	2022	Val Maira (Marmora Dronero, and Acceglio, specifically the hamlet of Chiappera)	600–1600	8 to 13	1300–1500	Various (25–75)	16 individuals, 3 dining establishments, and a culinary expert who has collaborated with local restaurants	Direct interviews, remote data collection, herbarium, and recipe books	Local economy is based on tourism, seasonal workers

## Data Availability

The data supporting this study’s findings are presented in this article.
